# Parental Misperception of Preschool Children’s Weight Status in China: Verbal Versus Visual Assessment and Associations with Feeding Practices

**DOI:** 10.3390/nu18213505

**Published:** 2026-10-25

**Authors:** Lan Shi, Yujia Chen, Xiaoxue Wei, Xinyi Song, Chenjun Wu, Fangge Qu, Xianqing Tang, Daqiao Zhu

**Affiliations:** 1 School of Nursing, Shanghai Jiao Tong University, Shanghai 201318, China; 2 Department of Global Public Health, Karolinska Institutet, SE-171 77 Stockholm, Sweden; 3 School of International Medical Technology, Nursing Department, Shanghai Sanda University, Shanghai 201209, China; 4 Department of Nursing, Shanghai Children’s Medical Center Affiliated to Shanghai Jiao Tong University School of Medicine, Shanghai 200127, China; 5 School of Nursing and Health Management, Shanghai University of Medicine & Health Sciences, Shanghai 201318, China; 6 Department of Children’s Disease Prevention, Jinyang Community Health Service Center, Shanghai 201206, China

**Keywords:** parental weight perception, weight underestimation, preschool children, verbal and visual assessment, feeding practices, childhood obesity

## Abstract

**Background:** Parental misperception of child weight status may influence feeding practices, with implications for early prevention of childhood obesity. This study compared verbal and visual assessments of parental perception and misperception of preschool children’s weight status and examined associations between method-specific parental weight underestimation and feeding practices. **Methods:** This cross-sectional study included 520 parent–child dyads in Shanghai, China. Parental perception was assessed using a verbal item and visual silhouettes and compared with objectively measured child weight status. Agreement with actual weight status, overall perception patterns, and differences in underestimation between methods were examined using kappa statistics, Bowker’s test, and McNemar’s test, respectively. Associations between method-specific underestimation and feeding practices were examined using multivariable linear regression with false discovery rate (FDR) correction. **Results:** Agreement with actual weight status was limited for both methods and lower for visual than verbal assessment (quadratic weighted κ = 0.21 vs. 0.37). Underestimation was more common with visual than verbal assessment in the overall sample (45.1% vs. 37.8%, *p* < 0.001), particularly among children with overweight or obesity (87.5% vs. 43.2%, *p* < 0.001). Verbal underestimation was associated with lower scores for content-restricted feeding (adjusted β = −0.159, 95% CI: −0.312 to −0.005, *p* = 0.043) and behavior-restricted feeding (adjusted β = −0.129, 95% CI: −0.252 to −0.007, *p* = 0.038), whereas visual underestimation was not consistently associated with feeding practices. However, neither verbal association remained statistically significant after FDR correction (both adjusted *p* = 0.203). **Conclusions:** Verbal and visual assessments showed different patterns of parental weight perception and misperception and different associations between parental underestimation and feeding practices. Appropriate tool selection may support parental recognition of children’s weight status and feeding-related guidance for early prevention of childhood obesity.

## 1. Introduction

Childhood overweight and obesity remain major public health challenges worldwide [1]. In China, between 1990 and 2022, obesity prevalence increased from 0.6% to 7.7% among girls and from 1.3% to 15.2% among boys [2]. Early prevention is particularly important during the preschool years, when children have limited autonomy over food choices and parents play a central role in shaping their dietary environments and feeding practices [3]. In this context, parental perceptions of children’s weight may be associated with weight-related concerns and feeding practices [4,5].

Parental perception of child weight encompasses judgments about a child’s weight status, body size, and body shape [6]. Yet parents’ perceptions of their children’s weight do not always align with objective anthropometric assessments [7]. Such discrepancies, referred to as parental weight misperception, may involve underestimation or overestimation. Underestimation is more common, particularly among parents of children with overweight or obesity and among parents of younger children [7,8,9].

Two commonly used approaches to assessing parental weight perception are verbal and visual assessments. Verbal assessments ask parents to classify their child’s weight status using categorical labels, whereas visual assessments ask parents to select a body silhouette or image that best represents their child’s body size and shape [8]. Although both approaches assess parental weight perception, they capture related but not necessarily equivalent aspects of this construct. Previous studies have compared the agreement of verbal and visual assessments with objectively measured weight status, but findings remain inconsistent. Some studies have reported better agreement for verbal assessments, others for visual assessments, and still others have found comparable performance [8,10,11,12,13,14,15,16,17,18]. However, studies directly comparing the agreement of verbal and visual assessments with objectively measured weight status among parents of Chinese preschool children remain limited.

One previous study among Mexican mothers extended these comparisons by examining associations between method-specific underestimation and feeding practices [11]. Because the two approaches elicit different types of judgments, they may identify different patterns of underestimation, which may also show different associations with feeding practices. However, evidence on associations between method-specific parental weight underestimation and feeding practices remains limited, particularly among Chinese preschool children.

Therefore, this study aimed to (1) compare verbal and visual assessments of parental perception and misperception of child weight status among Chinese preschool children and (2) examine associations between method-specific parental weight underestimation and parental feeding practices.

## 2. Methods

### 2.1. Study Design and Participants

This questionnaire-based cross-sectional study was conducted between December 2021 and January 2022 in eight kindergartens in Pudong New Area, Shanghai, China. Parent–child dyads were recruited using convenience sampling. Parents (mothers or fathers) of preschool children aged 3–6 years were eligible if they were the respondent completing the questionnaire and were the primary caregiver responsible for the child’s daily feeding. Parents were also required to have sufficient Chinese proficiency to complete the survey. Children were excluded if they had experienced conditions affecting appetite within the past month (e.g., fever, allergies, or gastrointestinal discomfort), or had psychiatric, neurological, or chronic physical conditions that could influence eating behavior. Parents who declined participation were not enrolled.

Of the participants in a larger research project, 568 parent–child dyads met the eligibility criteria for the present study. Questionnaires were excluded if they showed patterned responses, contained extreme values, or had more than 20% missing data in variables used in this study. After excluding 48 questionnaires, 520 were included in the analytic sample. Based on 18 study-relevant variables and a conventional criterion of 10–20 observations per variable, an approximate sample-size range of 180–360 participants was estimated. The final sample size of 520 exceeded this range. No formal a priori power calculation was performed for the present study. This study was approved by the Ethics Committee of Shanghai Jiao Tong University School of Medicine (SJUPN-201908; approval date: 3 December 2019).

### 2.2. Actual Child Weight Status

Children’s height (cm) and weight (kg) were measured by trained child healthcare professionals using standardized anthropometric procedures. BMI-for-age z-scores (BAZ) were calculated using WHO Anthro for children aged <5 years and WHO AnthroPlus for those aged ≥5 years. According to the 2007 WHO growth standards and references, child BAZ was treated as a categorical variable in this study: underweight (BAZ < −2), normal weight (−2 ≤ BAZ ≤ 1), or overweight/obesity (BAZ > 1) [19,20].

### 2.3. Parental Perception of Child Weight Status

Verbal and visual assessments were used to assess parental perception of child weight status.

For verbal assessment, one item from the Chinese version of the Child Feeding Questionnaire (C-CFQ) was used [21]. Parents were asked to classify their child’s current weight status as “markedly underweight”, “underweight”, “average”, “overweight”, or “markedly overweight”. Responses were categorized as perceived underweight (“markedly underweight” or “underweight”), perceived normal weight (“average”), or perceived overweight/obesity (“overweight” or “markedly overweight”). This item was taken from the perceived child weight dimension of the C-CFQ. In the validation study, this dimension showed a Cronbach’s α of 0.817 and a test–retest reliability of 0.936.

For visual assessment, a set of age- and sex-specific child body silhouettes developed by Warschburger and Kröller was used [22]. Parents were asked to select the silhouette that best represented their child’s current body size and shape. Responses were categorized as perceived underweight (first two silhouettes, corresponding to the 3rd and 10th BMI percentiles), perceived normal weight (middle three silhouettes, corresponding to the 25th, 50th, and 75th BMI percentiles), or perceived overweight/obesity (last two silhouettes, corresponding to the 90th and 97th BMI percentiles). The silhouettes were developed based on child anthropometric data and represent age- and sex-specific BMI percentile categories. Expert evaluation in the original study supported their content validity, with 86–91% of experts correctly ordering the silhouettes and perfect agreement in classifying silhouettes into underweight, normal-weight, and overweight categories. Before use in the present study, the silhouettes were reviewed by local child healthcare professionals involved in the study and considered contextually appropriate for assessing body size and shape among Chinese preschool children.

### 2.4. Parental Misperception of Child Weight Status

Parental perception of child weight status was compared with the child’s actual weight status. Perception was considered an accurate estimation when the perceived weight category matched the actual weight category. Underestimation was defined as perceiving the child’s weight status as lower than the actual category (i.e., perceiving a child with normal weight as underweight or a child with overweight/obesity as normal weight or underweight), whereas overestimation was defined as perceiving it as higher than the actual category.

### 2.5. Parental Feeding Practices

Parental feeding practices were assessed using selected items from the Chinese Preschoolers’ Caregivers’ Feeding Behavior Scale (CPCFBS) [23] and the C-CFQ [21]. A total of 27 items were used to measure six feeding practices: content-restricted feeding (CPCFBS), encourage healthy eating (CPCFBS), behavior-restricted feeding (CPCFBS), use of food as a reward (C-CFQ), forced feeding/pressure to eat (CPCFBS and C-CFQ), and supervise eating (CPCFBS). All items were rated on a 5-point Likert scale ranging from 1 (never) to 5 (always). Mean scores were calculated for each feeding practice, with higher scores indicating greater use of the corresponding practice. Both source instruments have been previously developed or validated among Chinese caregivers or parents of preschool children. For CPCFBS, the five dimensions used in the present study showed Cronbach’s α coefficients ranging from 0.65 to 0.88, split-half reliability ranging from 0.68 to 0.88, and test–retest reliability ranging from 0.65 to 0.84. For C-CFQ, the food-as-reward and pressure-to-eat dimensions showed Cronbach’s α coefficients of 0.695 and 0.747, respectively, and test–retest reliability coefficients of 0.873 and 0.934, respectively.

### 2.6. Sociodemographic Measures

Based on the previous literature [24] and the aims of the present study, the following sociodemographic characteristics were assessed through the questionnaire: child age, child sex, breastfeeding duration, only-child status, parental age, parental sex, parental height and weight, parental education level, and annual household income. Parental BMI was calculated from self-reported height and weight.

### 2.7. Statistical Analysis

Descriptive analyses were conducted for sociodemographic characteristics, parental perception and misperception of child weight status, and feeding practice scores. Continuous variables were presented as means and standard deviations (SDs), while categorical variables were summarized as counts and percentages.

Agreement between parental perception and children’s actual weight status was assessed separately for verbal and visual assessments using unweighted and quadratic weighted kappa statistics. Differences between verbal and visual assessments in overall perception patterns, including underestimation, accurate estimation, and overestimation, were examined using Bowker’s test. Differences between verbal and visual assessments in underestimation were examined using McNemar’s test in the overall sample and within strata of actual child weight status, comparing underestimation with non-underestimation (accurate estimation and overestimation combined).

To examine associations between parental weight misperception and feeding practices, linear regression analyses were conducted separately for verbal and visual assessments. Because of the small sample sizes in certain categories, children with actual underweight (*n* = 1) and cases of parental overestimation (*n* = 2 for visual assessment and *n* = 14 for verbal assessment) were excluded from the regression analyses. Therefore, parental weight misperception was operationalized as underestimation compared with accurate estimation in these models.

Bivariate linear regression analyses were first performed to obtain crude estimates. Multivariable linear regression models were then adjusted for six core covariates selected based on the prior literature and theoretical relevance: child age, child sex, child BAZ, parental BMI, parental education level, and annual household income [24]. Sensitivity analyses additionally adjusted for four further covariates: breastfeeding duration, only-child status, parental age, and parental sex. Model fit was summarized using R^2^, and multicollinearity was assessed using variance inflation factors (VIFs).

For the main adjusted regression models with six covariates, the Benjamini–Hochberg false discovery rate (BH-FDR) correction was applied to the *p*-values of the 12 main associations (six feeding practices × two assessment methods) to account for multiple comparisons, while a forest plot was used to visualize the corresponding regression coefficients and 95% confidence intervals (CIs).

Analyses of parental perception and misperception used observed complete data for the variables required in each specific analysis. Therefore, denominators varied across analyses. Complete-case analysis was applied separately for each regression model. To assess the potential influence of missing data, multiple imputation by chained equations was conducted as a sensitivity analysis for the main adjusted regression models, using 20 imputed datasets and pooling estimates according to Rubin’s rules. Analyses were conducted using R version 4.5.3 and RStudio version 2026.04.0+526. All statistical tests were two-sided, and a *p*-value < 0.05 was considered statistically significant.

## 3. Results

### 3.1. Sociodemographic Characteristics

A total of 520 parent–child dyads were included in the analysis (Table 1). The mean age of children was 58.01 months (SD = 10.58), and 47.3% were boys. Most children were of normal weight (82.2%), while 17.6% had overweight or obesity. Among respondent parents, 74.6% were mothers and 25.4% were fathers. The mean age of respondent parents was 36.55 years (SD = 4.76), and 28.3% had overweight or obesity. Overall, 75.6% of children had been breastfed for at least 6 months, and 54.3% were only-children. Most parents had a college education or above (86.3%), and 62.1% of households reported an annual income above 300,000 RMB.

### 3.2. Weight Perception and Misperception

Overall agreement between parental perception and children’s actual weight status was limited for both assessment methods and was lower for visual than verbal assessment (Table 2). Quadratic weighted kappa indicated fair agreement for verbal assessment (κ = 0.37, *p* < 0.001) and weaker agreement for visual assessment (κ = 0.21, *p* < 0.001). Unweighted kappa indicated fair agreement for verbal assessment (κ = 0.21, *p* < 0.001), whereas visual assessment did not show agreement beyond chance (κ = −0.01, *p* = 0.768).

As shown in Table 3, based on verbal assessment, 188 parents (37.8%) underestimated their child’s weight status, 295 (59.4%) accurately estimated it, and 14 (2.8%) overestimated it. Based on visual assessment, 224 parents (45.1%) underestimated their child’s weight status, 271 (54.5%) accurately estimated it, and 2 (0.4%) overestimated it. Overall perception patterns differed significantly between verbal and visual assessments (Bowker’s χ^2^ = 21.86, *df* = 2, *p* < 0.001). Underestimation was the predominant form of misperception for both assessment methods. Underestimation was also significantly more common with visual than verbal assessment in the overall sample (45.1% vs. 37.8%, McNemar’s χ^2^ = 10.94, *p* < 0.001).

As shown in Table 4, among children with normal weight, the proportion of underestimation did not differ significantly between verbal and visual assessments (36.8% vs. 36.0%, McNemar’s χ^2^ = 0.02, *p* = 0.884). In contrast, among children with overweight or obesity, underestimation was significantly more common with visual than verbal assessment (87.5% vs. 43.2%, McNemar’s χ^2^ = 36.23, *p* < 0.001).

### 3.3. Feeding Practices

Mean scores for the six feeding practices ranged from 2.97 to 4.15. The highest scores were observed for behavior-restricted feeding (4.15 ± 1.03), supervise eating (4.09 ± 0.92), and encourage healthy eating (4.08 ± 0.83), followed by content-restricted feeding (3.56 ± 1.02) and use of food as a reward (3.21 ± 1.03). The lowest score was observed for forced feeding/pressure to eat (2.97 ± 1.15).

### 3.4. Associations of Verbal and Visual Underestimation with Feeding Practices

Among the six feeding practices examined, verbal underestimation, compared with accurate verbal estimation, was associated with lower scores for content-restricted feeding and behavior-restricted feeding in crude models. These associations remained statistically significant after adjustment for the six core covariates: content-restricted feeding (β = −0.159, 95% CI: −0.312 to −0.005, *p* = 0.043) and behavior-restricted feeding (β = −0.129, 95% CI: −0.252 to −0.007, *p* = 0.038). Both associations also remained statistically significant in the additional covariate-adjusted analyses and multiple-imputation analyses. However, neither association remained statistically significant after BH-FDR correction for the 12 main adjusted comparisons (both adjusted *p* = 0.203).

**Figure 1:**
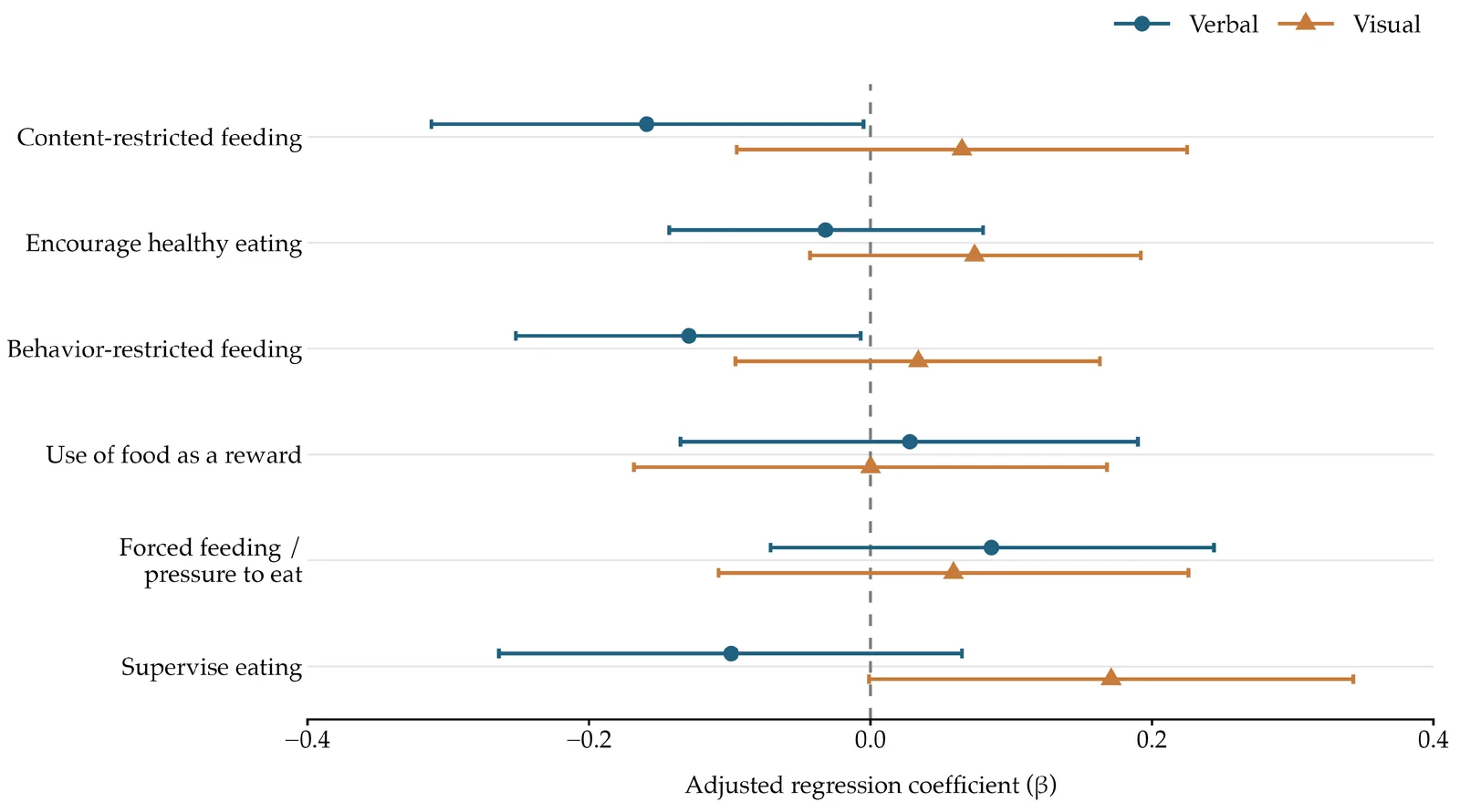
Forest plot of associations between parental weight underestimation and feeding practices in six-covariate adjusted models. Note. Points represent regression coefficients (β) comparing underestimation with accurate estimation (reference group); horizontal lines indicate 95% confidence intervals. The dashed vertical line indicates β = 0.

For visual assessment, underestimation was not consistently associated with any of the six feeding practices across models. The main adjusted regression results are presented in Table 5 and visualized in Figure 1, while results from the crude, additional covariate-adjusted, and multiple-imputation analyses are provided in the Supplementary Materials.

## 4. Discussion

This study provides new evidence that among Chinese preschool children parental weight perception and its relevance to feeding practices may differ by assessment method. Three key findings emerged. First, agreement with children’s actual weight status was limited for both methods, but lower for visual than verbal assessment. Second, underestimation was more common with visual than verbal assessment, particularly among children with overweight or obesity. Third, verbal underestimation was associated with lower scores for content- and behavior-restricted feeding, whereas visual underestimation was not consistently associated with feeding practices. However, neither verbal association remained statistically significant after correction for multiple testing. To our knowledge, this is among the first studies to compare verbal and visual assessments of parental weight perception in a Chinese preschool population and to further examine associations between method-specific parental underestimation and feeding practices. Together, these findings suggest that the choice of assessment method may be important not only for detecting parental underestimation but also for interpreting its relevance to feeding-related guidance in pediatric obesity prevention.

### 4.1. Verbal and Visual Assessments of Parental Weight Perception and Misperception

Consistent with the previous literature, the present study found that parents frequently underestimated children’s weight status, particularly among children with overweight or obesity [8,9]. However, previous studies comparing verbal and visual assessments have reported inconsistent findings, with some suggesting better agreement for verbal assessments [10,11], some reporting no clear differences between methods [12,13,14,15], and others suggesting better agreement for visual assessments [8,16,17,18]. The magnitude and direction of verbal–visual differences also vary across studies, likely reflecting differences in child age, cultural context, and assessment tools. Therefore, although agreement was lower for visual than verbal assessment in the present study, this finding should not be interpreted as evidence that one assessment method is inherently superior to the other. Rather, it suggests that verbal and visual assessments may capture different aspects of parental perception and involve different underlying processes, for which the dual-process theory may offer one possible explanation [25]. This theory distinguishes between relatively fast, intuitive processing and slower, more reflective processing.

Verbal assessment may involve more explicit, reflective evaluations of a child’s weight status. Parents may feel reluctant to describe their child as “overweight” because of the social stigma associated with weight-related labels, which may contribute to verbal underestimation [8,26,27]. In the Chinese context, however, this label-related stigma may be partly counterbalanced by cultural beliefs that associate a “chubby” child with being healthy, adequately nourished, or well cared for [4,28]. These beliefs may reduce parents’ reluctance to select a higher verbal weight category and thereby partly offset verbal underestimation.

In contrast, visual assessment may involve more intuitive, appearance-based perceptual judgments of a child’s body size and shape. According to visual normalization theory, as larger body sizes become increasingly common among children, repeated exposure to larger children may lead to parental visual adaptation and recalibration, shifting the threshold for identifying overweight upward and contributing to visual underestimation [29,30]. This mechanism may be particularly relevant in China, where childhood overweight and obesity have increased rapidly over the past two decades [2]. This broader epidemiological context was also reflected in the present sample, in which the proportion of child and parental overweight or obesity was not low. Such a context may contribute to a shift in parents’ visual reference frame for children’s body size and shape and may help explain why visual assessment showed lower agreement and greater underestimation than verbal assessment in the present study. This pattern is particularly relevant for pediatric obesity prevention because visual underestimation was most pronounced among children already classified as having overweight or obesity.

Measurement-related heterogeneity may also help explain inconsistent findings across previous studies regarding the relative performance of verbal and visual assessments [8,10,11,12,13,14,15,16,17,18]. Across studies, verbal tools vary in the wording of weight-status labels, such as “fat”, “overweight”, or “obese”, which may influence parents’ willingness to select higher weight categories. Visual tools also vary in image type, age and sex specificity, BMI validation, cultural appropriateness, and the number and granularity of response options.

Differences in the number and grouping of response options between verbal and visual assessments may have influenced the distribution of perceived weight categories and, consequently, estimates of agreement and underestimation. Moreover, the categories derived from verbal and visual assessments may not be fully equivalent to each other or directly correspond to WHO BMI-for-age categories. In addition, using three broad categories for both actual and perceived weight status may have obscured finer distinctions. Together, these considerations suggest that observed differences in agreement and underestimation may partly reflect measurement and classification differences, in addition to differences in the judgments elicited by verbal and visual assessments.

### 4.2. Method-Specific Parental Weight Underestimation and Feeding Practices

The current study further found that verbal underestimation was associated with lower scores for content- and behavior-restricted feeding, whereas visual underestimation was not consistently associated with feeding practices. However, neither verbal association remained statistically significant after correction for multiple testing. Consistent with the direction of our verbal findings, a U.S. study using verbal assessment alone found that caregiver underestimation of child weight was associated with lower restrictive feeding among preschool-aged children [31]. Similarly, a Mexican study comparing verbal and visual assessments found that verbal, but not visual, underestimation of children’s excess weight was associated with lower restrictive feeding. However, this association was observed among children aged 6–12 years, but not among those aged 2–5 years [11]. Together, these findings highlight the importance of considering how parental weight underestimation is assessed when examining its associations with feeding practices.

Verbal assessment may better reflect parents’ explicit cognitive interpretation of the child’s weight status, including consciously recognized beliefs and attitudes, and may therefore be more closely related to feeding-related intentions and behaviors [26,31,32]. When parents verbally underestimate their child’s weight, such as classifying a child with overweight or obesity as having normal weight, they may interpret the child’s weight status as normal or acceptable. In the Chinese context, verbal underestimation may also represent an explicit cognitive affirmation of the cultural belief that “chubby is healthy” [4,28]. This may reduce parents’ concern about, or dissatisfaction with, the child’s weight and lower their perceived need to regulate food content or eating behavior, which are relatively intentional feeding practices [33,34,35]. These processes may help explain the association between verbal underestimation and lower scores on these two feeding practices.

In contrast, visual underestimation may reflect a more intuitive and appearance-based perceptual judgment of the child’s body size and shape [26,36], which may not necessarily involve the same level of conscious interpretation or meaning assigned to the child’s weight status as verbal assessment. As a result, visual underestimation may be less likely to translate into weight-related concern, dissatisfaction, or feeding-related intentions, which may help explain its lack of consistent association with feeding practices in the present study.

Nevertheless, these proposed cognitive, perceptual, and cultural explanations are largely theory-informed. Direct empirical evidence for the underlying mechanisms remains limited, and these mechanisms were not examined in the present study.

### 4.3. Implications for Practice and Prevention

The findings of this study have implications for pediatric counseling and early childhood obesity prevention. Because parents often rely on their own perceptions rather than objective anthropometric measures to evaluate children’s weight status and make feeding-related decisions [4], the choice of assessment tool may influence how parental perceptions are identified and interpreted. Verbal assessment may be particularly informative when the aim is to support better parental recognition of child weight status and to guide discussions about feeding-related responses. In contrast, visual assessment may be useful when the aim is to identify parental underestimation, particularly among children with overweight or obesity. Therefore, the choice of assessment tool should depend on the specific purpose of assessment.

### 4.4. Limitations

Several methodological limitations should be considered when interpreting the findings of this study. First, the cross-sectional design precludes conclusions regarding causality or temporal relationships between parental weight underestimation and feeding practices. Residual confounding may also remain despite adjustment for measured covariates. Second, no formal a priori sample-size calculation was performed for the present study. Furthermore, the regression analyses included fewer participants than the overall sample, potentially reducing statistical precision and power to detect smaller associations. Some categories were sparsely represented and therefore excluded from the regression analyses. Only one child was classified as underweight, limiting insights into parental weight perception in this group. The small number of parental overestimations also limited our ability to examine associations with feeding practices across the full spectrum of parental weight misperception. Third, 48 questionnaires were excluded, primarily due to extensive missing responses, particularly in feeding-practice items, which may have introduced selection bias if excluded participants differed systematically from those retained. Although multiple-imputation results were consistent with the complete-case analyses among the 520 retained participants, bias due to missing data cannot be fully ruled out if the missing-at-random assumption was not met. Fourth, parental perception of child weight status and feeding practices were assessed using parent-reported questionnaires and may therefore be subject to social desirability bias. Finally, participants were recruited through convenience sampling with voluntary participation, which may have introduced selection bias. As participating families were recruited from a single district of Shanghai and most had relatively high educational and income levels, the findings may not be generalizable to other populations in China.

### 4.5. Future Directions

Future research should use longitudinal designs to examine whether method-specific parental underestimation predicts subsequent feeding practices. Further studies should also examine potential mechanisms underlying these associations, including parental concern about, dissatisfaction with, and cultural beliefs regarding child weight. In addition, future research should extend these assessments to more diverse caregivers, particularly fathers [37] and grandparents [38], to examine whether similar patterns of caregiver weight perception, misperception, and feeding practices are observed within Chinese family caregiving contexts.

## 5. Conclusions

Parental perception and misperception of preschool children’s weight status showed different patterns between verbal and visual assessments. Associations between parental underestimation and feeding practices also showed different patterns by assessment method. These findings suggest that verbal and visual assessments may capture different aspects of parental weight perception, with different relevance to feeding practices. Selecting appropriate assessment tools may help improve parental recognition of children’s weight status and inform feeding-related strategies for early prevention of childhood obesity.

## Figures and Tables

**Table 1:** Sociodemographic characteristics of participants (*n* = 520).

Variable	*n* (%) or Mean ± SD
Child characteristics	
Age (months) (*n* = 508)	58.01 ± 10.58
Sex	
Male	246 (47.3)
Female	274 (52.7)
Weight status (BAZ) (*n* = 505)	
Underweight (BAZ < −2)	1 (0.2)
Normal weight (−2 ≤ BAZ ≤ 1)	415 (82.2)
Overweight/obesity (BAZ > 1)	89 (17.6)
Breastfeeding duration (*n* = 517)	
<6 months	126 (24.4)
6–12 months	249 (48.2)
>12 months	142 (27.5)
Only child (*n* = 516)	
Yes	280 (54.3)
No	236 (45.7)
Parental characteristics	
Respondent	
Mother	388 (74.6)
Father	132 (25.4)
Age (years) (*n* = 506)	36.55 ± 4.76
Weight status (BMI) (*n* = 506)	
Underweight (<18.5 kg/m^2^)	41 (8.1)
Normal weight (18.5–23.9 kg/m^2^)	322 (63.6)
Overweight/obesity (≥24 kg/m^2^)	143 (28.3)
Education level (*n* = 517)	
High school or below	71 (13.7)
College or above	446 (86.3)
Annual household income (*n* = 485)	
≤300,000 RMB	184 (37.9)
>300,000 RMB	301 (62.1)

**Table 2:** Agreement between children’s actual weight status and parental perception by assessment method.

Actual Weight Status	Perceived Underweight	Perceived Normal Weight	Perceived Overweight/Obesity	Total
A. Verbal assessment				
Underweight	1 (100.0)	0 (0.0)	0 (0.0)	1
Normal weight	151 (36.5)	249 (60.1)	14 (3.4)	414
Overweight/obesity	4 (4.5)	35 (39.3)	50 (56.2)	89
Total	156 (31.0)	284 (56.3)	64 (12.7)	504
B. Visual assessment				
Underweight	1 (100.0)	0 (0.0)	0 (0.0)	1
Normal weight	147 (35.9)	260 (63.6)	2 (0.5)	409
Overweight/obesity	5 (5.7)	72 (81.8)	11 (12.5)	88
Total	153 (30.7)	332 (66.7)	13 (2.6)	498

Note. Values are *n* (row %). Verbal assessment analysis included participants with complete data on actual weight status and verbal perception (*n* = 504). Visual assessment analysis included participants with complete data on actual weight status and visual perception (*n* = 498). For verbal assessment, quadratic weighted κ = 0.37, *p* < 0.001; unweighted κ = 0.21, *p* < 0.001. For visual assessment, quadratic weighted κ = 0.21, *p* < 0.001; unweighted κ = −0.01, *p* = 0.768.

**Table 3:** Comparison of perception patterns between verbal and visual assessments.

	Visual Underestimation	Visual Accurate Estimation	Visual Overestimation	Total
Verbal underestimation	150	38	0	188
Verbal accurate estimation	74	220	1	295
Verbal overestimation	0	13	1	14
Total	224	271	2	497

Note. Analysis included participants with complete data on actual weight status, verbal perception, and visual perception (*n* = 497). Bowker’s test: χ^2^ = 21.86, *df* = 2, *p* < 0.001. McNemar’s test: overall sample, χ^2^ = 10.94, *p* < 0.001.

**Table 4:** Comparison of underestimation between verbal and visual assessments stratified by actual child weight status.

Actual Weight Status	Verbal Underestimation	Verbal Non-Underestimation	Visual Underestimation	Visual Non-Underestimation
Normal weight	150 (36.8)	258 (63.2)	147 (36.0)	261 (64.0)
Overweight/obesity	38 (43.2)	50 (56.8)	77 (87.5)	11 (12.5)

Note. Values are *n* (%). Analysis included participants with complete data on actual weight status, verbal perception, and visual perception (*n* = 497). The stratified comparison was restricted to children with normal weight or overweight/obesity because of the small underweight sample (*n* = 1). McNemar’s test: normal weight, χ^2^ = 0.02, *p* = 0.884; overweight/obesity, χ^2^ = 36.23, *p* < 0.001.

**Table 5:** Associations of verbal and visual underestimation with feeding practices in six-covariate adjusted models.

Statistic	Content- Restricted	Encourage Healthy Eating	Behavior- Restricted	Food as Reward	Forced Feeding	Supervise Eating
A. Verbal assessment						
β (SE)	−0.159 (0.078)	−0.032 (0.057)	−0.129 (0.062)	0.028 (0.083)	0.086 (0.080)	−0.099 (0.084)
95% CI	[−0.312, −0.005]	[−0.143, 0.080]	[−0.252, −0.007]	[−0.135, 0.190]	[−0.071, 0.244]	[−0.264, 0.065]
*p*	0.043	0.578	0.038	0.737	0.283	0.235
BH-adjusted *p*	0.203	0.730	0.203	0.804	0.565	0.564
N	446	449	449	449	449	449
R^2^	0.019	0.012	0.018	0.008	0.039	0.006
Maximum VIF	1.130	1.129	1.129	1.129	1.129	1.129
B. Visual assessment						
β (SE)	0.065 (0.081)	0.074 (0.060)	0.034 (0.066)	0.0001 (0.085)	0.059 (0.085)	0.171 (0.087)
95% CI	[−0.095, 0.225]	[−0.043, 0.192]	[−0.096, 0.163]	[−0.168, 0.168]	[−0.108, 0.226]	[−0.001, 0.343]
*p*	0.428	0.215	0.609	0.999	0.487	0.051
BH-adjusted *p*	0.730	0.564	0.730	0.999	0.730	0.203
N	457	460	460	460	460	460
R^2^	0.010	0.015	0.007	0.008	0.041	0.010
Maximum VIF	1.251	1.249	1.249	1.249	1.249	1.249

Note. Regression coefficients (β) compare parental underestimation with accurate estimation (reference group). Models were adjusted for child age, child sex, child BAZ, parental BMI, parental education level, and annual household income. BH-adjusted *p*-values were calculated across the 12 associations shown. N represents the complete-case sample size for each model. Abbreviations: SE, standard error; CI, confidence interval; BH, Benjamini–Hochberg; R^2^, coefficient of determination; VIF, variance inflation factor.

## Data Availability

The data that support the findings of this study are available from the corresponding author upon reasonable request.

## Supplementary Materials

The following supporting information can be downloaded at: https://www.mdpi.com/article/10.3390/nu18213505/s1, Table S1: Crude associations between weight underestimation and feeding practices; Table S2: Main adjusted regression models (six covariates); Table S3: Additional adjusted regression models (ten covariates); Table S4: Complete-case and multiple-imputation estimates for the main adjusted models.
