# Supplementary material for: Parental Misperception of Preschool Children’s Weight Status in China: Verbal Versus Visual Assessment and Associations with Feeding Practices
Source: Nutrients. 2026 Oct 25;18(21):3505. doi: 10.3390/nu18213505 (PMC13764757; doi:10.3390/nu18213505)
Supplement: Supplementary file 1 [file nutrients-18-03505-s001.zip › nutrients-4615404-supplementary.pdf]

Supplementary Tables

Associations of parental verbal and visual weight underestimation with feeding practices

General Note. Across Tables S1–S4, parental weight underestimation was compared with accurate estimation (reference group). The child with underweight and cases of parental overestimation were excluded. β, unstandardized regression coefficient; SE, standard error; CI, confidence interval; N, sample size; R^2, coefficient of determination; VIF, variance inflation factor. Reported p-values were not adjusted for multiple testing.

Table S1. Crude associations between weight underestimation and feeding practices

Feeding practice	Assessment	β (SE)	95% CI	p	N
Content-restricted	Verbal	-0.167 (0.072)	[-0.307, -0.026]	0.020	486
Content-restricted	Visual	0.032 (0.069)	[-0.103, 0.168]	0.641	492
Encourage healthy eating	Verbal	-0.033 (0.054)	[-0.139, 0.073]	0.542	489
Encourage healthy eating	Visual	0.036 (0.052)	[-0.067, 0.139]	0.495	495
Behavior-restricted	Verbal	-0.123 (0.059)	[-0.238, -0.007]	0.037	489
Behavior-restricted	Visual	-0.013 (0.058)	[-0.126, 0.100]	0.819	495
Food as reward	Verbal	0.021 (0.076)	[-0.128, 0.170]	0.778	489
Food as reward	Visual	-0.032 (0.073)	[-0.175, 0.111]	0.660	495
Forced feeding	Verbal	0.073 (0.076)	[-0.076, 0.221]	0.337	489
Forced feeding	Visual	-0.008 (0.074)	[-0.154, 0.137]	0.909	495
Supervise eating	Verbal	-0.111 (0.078)	[-0.265, 0.043]	0.159	489
Supervise eating	Visual	0.078 (0.076)	[-0.071, 0.228]	0.304	495

Note. Separate univariable linear regression models were fitted for each assessment method and feeding practice.

Table S2. Main adjusted regression models (six covariates)

Panel A. Verbal assessment

Predictor	Content-restricted	Encourage healthy eating	Behavior-restricted	Food as reward	Forced feeding	Supervise eating
Intercept	3.667 (0.221)<br>[3.233, 4.101]; p<0.001	4.308 (0.161)<br>[3.993, 4.624]; p<0.001	4.317 (0.176)<br>[3.971, 4.663]; p<0.001	3.498 (0.234)<br>[3.038, 3.958]; p<0.001	3.526 (0.227)<br>[3.079, 3.972]; p<0.001	4.224 (0.236)<br>[3.759, 4.688]; p<0.001
**Weight underestimation**	-0.159 (0.078)<br>[-0.312, -0.005]; p=0.043	-0.032 (0.057)<br>[-0.143, 0.080]; p=0.578	-0.129 (0.062)<br>[-0.252, -0.007]; p=0.038	0.028 (0.083)<br>[-0.135, 0.190]; p=0.737	0.086 (0.080)<br>[-0.071, 0.244]; p=0.283	-0.099 (0.084)<br>[-0.264, 0.065]; p=0.235
Child age (months)	-0.001 (0.004)<br>[-0.007, 0.006]; p=0.883	-0.004 (0.003)<br>[-0.009, 0.001]; p=0.130	-0.002 (0.003)<br>[-0.007, 0.004]; p=0.584	-0.005 (0.004)<br>[-0.012, 0.003]; p=0.220	-0.010 (0.004)<br>[-0.017, -0.003]; p=0.006	-0.001 (0.004)<br>[-0.009, 0.006]; p=0.768
Female child	-0.032 (0.076)<br>[-0.181, 0.117]; p=0.673	0.046 (0.055)<br>[-0.062, 0.154]; p=0.406	0.036 (0.060)<br>[-0.083, 0.154]; p=0.555	-0.028 (0.080)<br>[-0.186, 0.129]; p=0.725	-0.076 (0.078)<br>[-0.229, 0.077]; p=0.328	-0.034 (0.081)<br>[-0.193, 0.125]; p=0.677
Child overweight/obesity	-0.032 (0.097)<br>[-0.223, 0.159]; p=0.740	-0.024 (0.071)<br>[-0.164, 0.115]; p=0.731	-0.042 (0.078)<br>[-0.195, 0.111]; p=0.589	-0.057 (0.103)<br>[-0.260, 0.146]; p=0.580	-0.088 (0.100)<br>[-0.285, 0.110]; p=0.384	-0.083 (0.104)<br>[-0.288, 0.123]; p=0.430
Parent underweight	-0.054 (0.139)<br>[-0.327, 0.220]; p=0.700	-0.111 (0.102)<br>[-0.310, 0.089]; p=0.276	-0.091 (0.111)<br>[-0.310, 0.128]; p=0.414	0.015 (0.148)<br>[-0.276, 0.305]; p=0.921	0.198 (0.144)<br>[-0.084, 0.480]; p=0.168	0.041 (0.149)<br>[-0.253, 0.334]; p=0.786
Parent overweight/obesity	0.024 (0.086)<br>[-0.146, 0.193]; p=0.785	-0.023 (0.063)<br>[-0.146, 0.100]; p=0.716	-0.095 (0.069)<br>[-0.230, 0.040]; p=0.169	0.051 (0.091)<br>[-0.129, 0.230]; p=0.580	0.188 (0.089)<br>[0.014, 0.362]; p=0.034	0.028 (0.092)<br>[-0.153, 0.209]; p=0.763
Parent education: high school or below	-0.196 (0.111)<br>[-0.414, 0.022]; p=0.078	-0.024 (0.081)<br>[-0.182, 0.135]; p=0.769	0.001 (0.088)<br>[-0.173, 0.174]; p=0.993	-0.127 (0.117)<br>[-0.358, 0.103]; p=0.278	-0.157 (0.114)<br>[-0.381, 0.067]; p=0.168	-0.019 (0.118)<br>[-0.251, 0.214]; p=0.875
Household income ≤ RMB 300,000	0.092 (0.081)<br>[-0.068, 0.251]; p=0.260	0.036 (0.059)<br>[-0.080, 0.152]; p=0.545	0.015 (0.065)<br>[-0.112, 0.142]; p=0.814	0.049 (0.086)<br>[-0.119, 0.218]; p=0.566	-0.003 (0.083)<br>[-0.167, 0.161]; p=0.972	0.009 (0.087)<br>[-0.161, 0.180]; p=0.916
N	446	449	449	449	449	449
R^2	0.019	0.012	0.018	0.008	0.039	0.006
Maximum VIF	1.130	1.129	1.129	1.129	1.129	1.129

Table S2 (continued).

Panel B. Visual assessment

Predictor	Content-restricted	Encourage healthy eating	Behavior-restricted	Food as reward	Forced feeding	Supervise eating
Intercept	3.495 (0.213) [3.076, 3.913]; p<0.001	4.184 (0.157) [3.876, 4.491]; p<0.001	4.232 (0.173) [3.893, 4.571]; p<0.001	3.503 (0.223) [3.063, 3.942]; p<0.001	3.569 (0.222) [3.132, 4.005]; p<0.001	4.028 (0.229) [3.579, 4.478]; p<0.001
**Weight underestimation**	0.065 (0.081) [-0.095, 0.225]; p=0.428	0.074 (0.060) [-0.043, 0.192]; p=0.215	0.034 (0.066) [-0.096, 0.163]; p=0.609	0.0001 (0.085) [-0.168, 0.168]; p=0.999	0.059 (0.085) [-0.108, 0.226]; p=0.487	0.171 (0.087) [-0.001, 0.343]; p=0.051
Child age (months)	0.001 (0.003) [-0.006, 0.007]; p=0.878	-0.003 (0.003) [-0.008, 0.002]; p=0.269	-0.002 (0.003) [-0.007, 0.004]; p=0.563	-0.004 (0.004) [-0.011, 0.003]; p=0.219	-0.010 (0.004) [-0.017, -0.003]; p=0.004	0.0002 (0.004) [-0.007, 0.007]; p=0.956
Female child	0.002 (0.074) [-0.143, 0.147]; p=0.980	0.065 (0.054) [-0.042, 0.171]; p=0.232	0.048 (0.060) [-0.070, 0.165]; p=0.423	-0.052 (0.077) [-0.205, 0.100]; p=0.498	-0.116 (0.077) [-0.267, 0.035]; p=0.133	-0.023 (0.079) [-0.178, 0.133]; p=0.774
Child overweight/obesity	-0.066 (0.106) [-0.274, 0.143]; p=0.535	-0.055 (0.078) [-0.208, 0.099]; p=0.485	-0.057 (0.086) [-0.226, 0.112]; p=0.506	-0.050 (0.112) [-0.269, 0.169]; p=0.653	-0.111 (0.111) [-0.328, 0.107]; p=0.319	-0.168 (0.114) [-0.392, 0.057]; p=0.143
Parent underweight	-0.083 (0.138) [-0.354, 0.189]; p=0.550	-0.123 (0.102) [-0.323, 0.077]; p=0.229	-0.103 (0.112) [-0.323, 0.118]; p=0.361	0.025 (0.145) [-0.261, 0.310]; p=0.866	0.205 (0.144) [-0.079, 0.489]; p=0.157	0.001 (0.149) [-0.291, 0.293]; p=0.993
Parent overweight/obesity	0.034 (0.084) [-0.131, 0.198]; p=0.688	-0.038 (0.061) [-0.159, 0.083]; p=0.534	-0.061 (0.068) [-0.194, 0.072]; p=0.368	0.051 (0.088) [-0.122, 0.223]; p=0.565	0.184 (0.087) [0.012, 0.355]; p=0.036	0.030 (0.090) [-0.146, 0.207]; p=0.735
Parent education: high school or below	-0.200 (0.109) [-0.415, 0.014]; p=0.067	-0.021 (0.080) [-0.177, 0.136]; p=0.796	-0.0002 (0.088) [-0.173, 0.173]; p=0.998	-0.122 (0.114) [-0.346, 0.102]; p=0.286	-0.154 (0.113) [-0.376, 0.069]; p=0.176	-0.042 (0.117) [-0.271, 0.188]; p=0.722
Household income ≤ RMB 300,000	0.095 (0.079) [-0.060, 0.250]; p=0.230	0.053 (0.058) [-0.061, 0.167]; p=0.362	0.015 (0.064) [-0.110, 0.141]; p=0.810	0.050 (0.083) [-0.112, 0.213]; p=0.544	-0.003 (0.082) [-0.165, 0.159]; p=0.970	0.027 (0.085) [-0.140, 0.193]; p=0.750
N	457	460	460	460	460	460
R^2	0.010	0.015	0.007	0.008	0.041	0.010
Maximum VIF	1.251	1.249	1.249	1.249	1.249	1.249

Note. Models were adjusted for six core covariates: child age, child sex, child weight status, parental BMI, parental education, and household income. Reference categories were male child, normal child weight, normal parental BMI, college education or above, and household income > RMB 300,000. Maximum VIF was calculated for model-matrix dummy variables.

Table S3. Additional adjusted regression models (ten covariates)

Panel A. Verbal assessment

Predictor	Content-restricted	Encourage healthy eating	Behavior-restricted	Food as reward	Forced feeding	Supervise eating
Intercept	3.757 (0.369)<br>[3.032, 4.481]; p<0.001	3.970 (0.264)<br>[3.452, 4.489]; p<0.001	4.469 (0.294)<br>[3.890, 5.048]; p<0.001	3.060 (0.388)<br>[2.298, 3.822]; p<0.001	3.285 (0.377)<br>[2.543, 4.027]; p<0.001	4.289 (0.394)<br>[3.515, 5.063]; p<0.001
**Weight underestimation**	-0.167 (0.079)<br>[-0.321, -0.012]; p=0.034	-0.030 (0.056)<br>[-0.141, 0.080]; p=0.588	-0.129 (0.063)<br>[-0.252, -0.005]; p=0.041	0.032 (0.083)<br>[-0.130, 0.194]; p=0.698	0.094 (0.080)<br>[-0.064, 0.252]; p=0.244	-0.089 (0.084)<br>[-0.254, 0.076]; p=0.288
Child age (months)	-0.0001 (0.004)<br>[-0.007, 0.007]; p=0.971	-0.004 (0.003)<br>[-0.009, 0.001]; p=0.125	-0.001 (0.003)<br>[-0.006, 0.005]; p=0.815	-0.005 (0.004)<br>[-0.013, 0.002]; p=0.153	-0.010 (0.004)<br>[-0.017, -0.003]; p=0.009	-0.0001 (0.004)<br>[-0.008, 0.008]; p=0.978
Female child	-0.053 (0.077)<br>[-0.203, 0.098]; p=0.491	0.019 (0.055)<br>[-0.089, 0.127]; p=0.728	0.018 (0.061)<br>[-0.103, 0.138]; p=0.774	-0.056 (0.081)<br>[-0.214, 0.103]; p=0.490	-0.085 (0.078)<br>[-0.239, 0.069]; p=0.281	-0.047 (0.082)<br>[-0.208, 0.114]; p=0.567
Child overweight/obesity	-0.059 (0.098)<br>[-0.253, 0.134]; p=0.547	-0.049 (0.071)<br>[-0.188, 0.090]; p=0.490	-0.073 (0.079)<br>[-0.228, 0.082]; p=0.354	-0.055 (0.104)<br>[-0.259, 0.149]; p=0.598	-0.076 (0.101)<br>[-0.275, 0.122]; p=0.451	-0.085 (0.105)<br>[-0.293, 0.122]; p=0.419
Parent underweight	-0.061 (0.143)<br>[-0.343, 0.220]; p=0.668	-0.143 (0.103)<br>[-0.345, 0.059]; p=0.165	-0.111 (0.115)<br>[-0.336, 0.114]; p=0.334	0.027 (0.151)<br>[-0.269, 0.324]; p=0.856	0.147 (0.147)<br>[-0.142, 0.436]; p=0.317	0.001 (0.153)<br>[-0.300, 0.303]; p=0.992
Parent overweight/obesity	0.072 (0.092)<br>[-0.108, 0.253]; p=0.432	-0.006 (0.066)<br>[-0.135, 0.123]; p=0.927	-0.069 (0.073)<br>[-0.213, 0.074]; p=0.343	0.045 (0.096)<br>[-0.144, 0.235]; p=0.637	0.168 (0.094)<br>[-0.016, 0.352]; p=0.073	0.065 (0.098)<br>[-0.127, 0.257]; p=0.507
Parent education: high school or below	-0.208 (0.112)<br>[-0.428, 0.013]; p=0.064	-0.007 (0.080)<br>[-0.164, 0.150]; p=0.930	-0.005 (0.089)<br>[-0.181, 0.170]; p=0.951	-0.105 (0.118)<br>[-0.336, 0.126]; p=0.373	-0.144 (0.115)<br>[-0.369, 0.081]; p=0.208	-0.023 (0.120)<br>[-0.258, 0.212]; p=0.846
Household income ≤ RMB 300,000	0.084 (0.082)<br>[-0.077, 0.245]; p=0.304	0.030 (0.058)<br>[-0.085, 0.145]; p=0.607	0.015 (0.065)<br>[-0.113, 0.143]; p=0.819	0.027 (0.086)<br>[-0.142, 0.195]; p=0.756	-0.021 (0.084)<br>[-0.185, 0.143]; p=0.801	-0.010 (0.087)<br>[-0.181, 0.161]; p=0.908
Breastfeeding: 6–12 months	0.034 (0.093)<br>[-0.150, 0.218]; p=0.716	0.123 (0.067)<br>[-0.008, 0.254]; p=0.066	0.094 (0.074)<br>[-0.052, 0.241]; p=0.206	-0.001 (0.098)<br>[-0.193, 0.192]; p=0.996	-0.067 (0.095)<br>[-0.255, 0.121]; p=0.483	-0.046 (0.100)<br>[-0.241, 0.150]; p=0.647
Breastfeeding: >12 months	0.113 (0.106)<br>[-0.095, 0.322]; p=0.285	0.262 (0.076)<br>[0.113, 0.411]; p<0.001	0.206 (0.085)<br>[0.039, 0.372]; p=0.016	0.228 (0.112)<br>[0.009, 0.448]; p=0.041	0.103 (0.109)<br>[-0.111, 0.316]; p=0.344	0.129 (0.113)<br>[-0.094, 0.352]; p=0.256
Only child: yes	-0.039 (0.079)<br>[-0.195, 0.116]; p=0.620	0.125 (0.057)<br>[0.014, 0.237]; p=0.028	0.003 (0.063)<br>[-0.121, 0.127]; p=0.961	0.128 (0.083)<br>[-0.036, 0.292]; p=0.125	0.130 (0.081)<br>[-0.030, 0.289]; p=0.110	0.037 (0.085)<br>[-0.129, 0.204]; p=0.660
Parent age (years)	-0.002 (0.008)<br>[-0.019, 0.014]; p=0.790	0.006 (0.006)<br>[-0.006, 0.017]; p=0.350	-0.008 (0.007)<br>[-0.021, 0.005]; p=0.247	0.011 (0.009)<br>[-0.006, 0.029]; p=0.194	0.005 (0.009)<br>[-0.011, 0.022]; p=0.527	-0.003 (0.009)<br>[-0.021, 0.014]; p=0.729
Father respondent	-0.167 (0.093)<br>[-0.349, 0.016]; p=0.074	-0.147 (0.067)<br>[-0.278, -0.017]; p=0.027	-0.050 (0.074)<br>[-0.196, 0.096]; p=0.500	-0.125 (0.098)<br>[-0.317, 0.067]; p=0.202	-0.047 (0.095)<br>[-0.234, 0.140]; p=0.624	-0.143 (0.099)<br>[-0.338, 0.052]; p=0.151
N	441	444	444	444	444	444
R^2	0.032	0.061	0.037	0.034	0.052	0.019
Maximum VIF	1.574	1.567	1.567	1.567	1.567	1.567

Table S3 (continued).

Panel B. Visual assessment

Predictor	Content-restricted	Encourage healthy eating	Behavior-restricted	Food as reward	Forced feeding	Supervise eating
Intercept	3.442 (0.368)<br>[2.719, 4.166]; p<0.001	3.748 (0.266)<br>[3.224, 4.271]; p<0.001	4.356 (0.298)<br>[3.769, 4.942]; p<0.001	3.078 (0.383)<br>[2.326, 3.831]; p<0.001	3.340 (0.381)<br>[2.591, 4.088]; p<0.001	4.030 (0.394)<br>[3.256, 4.804]; p<0.001
**Weight underestimation**	0.048 (0.082)<br>[-0.114, 0.210]; p=0.560	0.064 (0.060)<br>[-0.053, 0.181]; p=0.282	0.033 (0.067)<br>[-0.099, 0.164]; p=0.626	-0.004 (0.086)<br>[-0.173, 0.164]; p=0.959	0.070 (0.085)<br>[-0.098, 0.238]; p=0.412	0.175 (0.088)<br>[0.002, 0.348]; p=0.047
Child age (months)	0.001 (0.004)<br>[-0.006, 0.008]; p=0.845	-0.003 (0.003)<br>[-0.008, 0.002]; p=0.266	-0.001 (0.003)<br>[-0.006, 0.005]; p=0.824	-0.005 (0.004)<br>[-0.012, 0.002]; p=0.178	-0.010 (0.004)<br>[-0.017, -0.003]; p=0.008	0.001 (0.004)<br>[-0.006, 0.009]; p=0.759
Female child	-0.015 (0.075)<br>[-0.163, 0.132]; p=0.840	0.041 (0.054)<br>[-0.066, 0.147]; p=0.452	0.031 (0.061)<br>[-0.088, 0.151]; p=0.608	-0.078 (0.078)<br>[-0.232, 0.075]; p=0.316	-0.124 (0.078)<br>[-0.276, 0.029]; p=0.111	-0.036 (0.080)<br>[-0.194, 0.122]; p=0.654
Child overweight/obesity	-0.078 (0.107)<br>[-0.289, 0.133]; p=0.469	-0.066 (0.078)<br>[-0.218, 0.087]; p=0.399	-0.082 (0.087)<br>[-0.253, 0.090]; p=0.350	-0.041 (0.112)<br>[-0.260, 0.179]; p=0.716	-0.098 (0.111)<br>[-0.317, 0.121]; p=0.378	-0.165 (0.115)<br>[-0.391, 0.061]; p=0.153
Parent underweight	-0.088 (0.142)<br>[-0.368, 0.192]; p=0.536	-0.148 (0.103)<br>[-0.351, 0.055]; p=0.152	-0.127 (0.116)<br>[-0.355, 0.100]; p=0.273	0.041 (0.149)<br>[-0.251, 0.333]; p=0.782	0.156 (0.148)<br>[-0.135, 0.447]; p=0.292	-0.036 (0.153)<br>[-0.336, 0.265]; p=0.816
Parent overweight/obesity	0.072 (0.089)<br>[-0.103, 0.247]; p=0.420	-0.034 (0.064)<br>[-0.160, 0.093]; p=0.602	-0.035 (0.072)<br>[-0.177, 0.106]; p=0.623	0.037 (0.093)<br>[-0.145, 0.218]; p=0.693	0.148 (0.092)<br>[-0.033, 0.329]; p=0.109	0.055 (0.095)<br>[-0.132, 0.242]; p=0.560
Parent education: high school or below	-0.201 (0.110)<br>[-0.418, 0.016]; p=0.069	0.004 (0.080)<br>[-0.152, 0.161]; p=0.956	-0.002 (0.089)<br>[-0.177, 0.174]; p=0.984	-0.095 (0.114)<br>[-0.320, 0.130]; p=0.409	-0.138 (0.114)<br>[-0.362, 0.086]; p=0.227	-0.042 (0.118)<br>[-0.273, 0.190]; p=0.724
Household income ≤ RMB 300,000	0.088 (0.080)<br>[-0.068, 0.245]; p=0.270	0.051 (0.058)<br>[-0.062, 0.164]; p=0.377	0.015 (0.064)<br>[-0.111, 0.142]; p=0.813	0.032 (0.083)<br>[-0.130, 0.195]; p=0.695	-0.019 (0.082)<br>[-0.180, 0.143]; p=0.822	0.010 (0.085)<br>[-0.157, 0.177]; p=0.906
Breastfeeding: 6–12 months	0.030 (0.092)<br>[-0.151, 0.211]; p=0.745	0.101 (0.066)<br>[-0.030, 0.232]; p=0.130	0.067 (0.075)<br>[-0.079, 0.214]; p=0.367	-0.005 (0.096)<br>[-0.193, 0.183]; p=0.957	-0.097 (0.095)<br>[-0.284, 0.090]; p=0.307	-0.064 (0.098)<br>[-0.257, 0.130]; p=0.519
Breastfeeding: >12 months	0.102 (0.103)<br>[-0.100, 0.305]; p=0.320	0.229 (0.075)<br>[0.082, 0.376]; p=0.002	0.168 (0.084)<br>[0.004, 0.332]; p=0.045	0.197 (0.107)<br>[-0.014, 0.408]; p=0.067	0.062 (0.107)<br>[-0.148, 0.271]; p=0.564	0.108 (0.110)<br>[-0.109, 0.325]; p=0.328
Only child: yes	-0.011 (0.079)<br>[-0.165, 0.144]; p=0.891	0.138 (0.057)<br>[0.026, 0.250]; p=0.016	0.023 (0.064)<br>[-0.102, 0.149]; p=0.714	0.146 (0.082)<br>[-0.015, 0.307]; p=0.075	0.148 (0.081)<br>[-0.012, 0.308]; p=0.069	0.047 (0.084)<br>[-0.118, 0.213]; p=0.574
Parent age (years)	0.002 (0.008)<br>[-0.015, 0.018]; p=0.848	0.008 (0.006)<br>[-0.004, 0.020]; p=0.171	-0.007 (0.007)<br>[-0.020, 0.007]; p=0.314	0.010 (0.009)<br>[-0.007, 0.027]; p=0.244	0.004 (0.009)<br>[-0.013, 0.022]; p=0.611	-0.001 (0.009)<br>[-0.019, 0.017]; p=0.892
Father respondent	-0.152 (0.090)<br>[-0.329, 0.026]; p=0.093	-0.121 (0.065)<br>[-0.249, 0.008]; p=0.066	-0.045 (0.073)<br>[-0.189, 0.099]; p=0.543	-0.083 (0.094)<br>[-0.268, 0.102]; p=0.378	0.013 (0.094)<br>[-0.171, 0.197]; p=0.888	-0.114 (0.097)<br>[-0.305, 0.076]; p=0.238
N	452	455	455	455	455	455
R^2	0.019	0.056	0.021	0.031	0.053	0.022
Maximum VIF	1.584	1.577	1.577	1.577	1.577	1.577

Note. Models included the six core covariates in Table S2 and four additional covariates: breastfeeding duration (reference: <6 months), only-child status (reference: No), parental age, and parental sex (reference: Mother). Maximum VIF was calculated for model-matrix dummy variables.

Table S4. Complete-case and multiple-imputation estimates for the main adjusted models

Feeding practice	Assessment	Analysis	β (SE)	95% CI	p	N
Content-restricted	Verbal	Complete case	-0.159 (0.078)	[-0.312, -0.005]	0.043	446
Content-restricted	Verbal	Multiple imputation	-0.172 (0.074)	[-0.318, -0.026]	0.021	505
Content-restricted	Visual	Complete case	0.065 (0.081)	[-0.095, 0.225]	0.428	457
Content-restricted	Visual	Multiple imputation	0.078 (0.080)	[-0.080, 0.235]	0.331	517
Encourage healthy eating	Verbal	Complete case	-0.032 (0.057)	[-0.143, 0.080]	0.578	449
Encourage healthy eating	Verbal	Multiple imputation	-0.021 (0.056)	[-0.130, 0.088]	0.707	505
Encourage healthy eating	Visual	Complete case	0.074 (0.060)	[-0.043, 0.192]	0.215	460
Encourage healthy eating	Visual	Multiple imputation	0.056 (0.059)	[-0.060, 0.172]	0.343	517
Behavior-restricted	Verbal	Complete case	-0.129 (0.062)	[-0.252, -0.007]	0.038	449
Behavior-restricted	Verbal	Multiple imputation	-0.123 (0.060)	[-0.241, -0.004]	0.042	505
Behavior-restricted	Visual	Complete case	0.034 (0.066)	[-0.096, 0.163]	0.609	460
Behavior-restricted	Visual	Multiple imputation	-0.002 (0.063)	[-0.127, 0.123]	0.973	517
Food as reward	Verbal	Complete case	0.028 (0.083)	[-0.135, 0.190]	0.737	449
Food as reward	Verbal	Multiple imputation	0.028 (0.077)	[-0.123, 0.179]	0.714	505
Food as reward	Visual	Complete case	0.0001 (0.085)	[-0.168, 0.168]	0.999	460
Food as reward	Visual	Multiple imputation	-0.002 (0.079)	[-0.158, 0.154]	0.982	517
Forced feeding	Verbal	Complete case	0.086 (0.080)	[-0.071, 0.244]	0.283	449
Forced feeding	Verbal	Multiple imputation	0.090 (0.077)	[-0.061, 0.241]	0.241	505
Forced feeding	Visual	Complete case	0.059 (0.085)	[-0.108, 0.226]	0.487	460
Forced feeding	Visual	Multiple imputation	0.046 (0.081)	[-0.112, 0.205]	0.566	517
Supervise eating	Verbal	Complete case	-0.099 (0.084)	[-0.264, 0.065]	0.235	449
Supervise eating	Verbal	Multiple imputation	-0.117 (0.081)	[-0.277, 0.042]	0.148	505
Supervise eating	Visual	Complete case	0.171 (0.087)	[-0.001, 0.343]	0.051	460
Supervise eating	Visual	Multiple imputation	0.124 (0.086)	[-0.045, 0.293]	0.151	517

Note. Models included the six core covariates in Table S2. Multiple imputation used 20 imputed datasets, with estimates pooled using Rubin's rules. For multiple imputation, N represents the assessment-specific dataset size.
